# Reconfigurable and tunable twisted light laser

**DOI:** 10.1038/s41598-018-29868-8

**Published:** 2018-07-30

**Authors:** Nan Zhou, Jun Liu, Jian Wang

**Affiliations:** Wuhan National Laboratory for Optoelectronics, School of Optical and Electronic Information, Huazhong University of Science and Technology, Wuhan, 430074 Hubei China

## Abstract

Twisted light, having a helical spatial phase structure and carrying orbital angular momentum (OAM), has given rise to many developments ranging from optical manipulation to optical communications. The laser excitation of twisted light in a reconfigurable and tunable way is of great interest. Here, we propose and experimentally demonstrate an OAM reconfigurable and wavelength tunable twisted light laser with achievable high-order OAM modes on a hybrid free-space and fiber platform. The excited twisted light laser is enabled by a ring resonator incorporating spatial light modulators (SLMs) and bandpass filter (BPF). By appropriately switching the phase pattern loaded onto SLMs and adjusting the BPF, twisted light laser with reconfigurable OAM and tunable wavelength is implemented. In the experiment, the OAM value is varied from −10 to +10 and the wavelength is adjusted from 1530 to 1565 nm covering the whole C band. The obtained results indicate successful implementation of a reconfigurable and tunable twisted light laser with favorable operation performance. Reconfigurable and tunable twisted light laser may open up new perspectives to more extensive OAM-enabled applications with improved flexibility and robustness.

## Introduction

In recent years, the space domain of lightwaves has gained great interest in diverse applications. Exploiting the spatial structure of lightwaves enables a new kind of light beam called structured light beyond the well-known Gaussian light beam. In general, structured light manifests distinct spatial amplitude/phase/polarization distribution^1–5^. Among different kinds of structured light, a promising one is called twisted light, featuring a helical phasefront, a phase singularity, and a doughnut intensity profile. Since the early recognition in 1992 by Allen and co-workers that lightwaves comparising a helical phasefront exp(i*lφ*) have an orbital angular momentum (OAM) of *lħ* per photon (*l*: topological charge, *φ*: azimuthal angle, *ħ*: reduced Plank’s constant)^2^, OAM-carrying twisted light has given rise to many developments in optical manipulation, tweezer, microscopy, imaging, metrology, astronomy and quantum information processing^6–16^. Very recently, twisted light has also seen its potential applications in free-space, fiber-based and underwater optical communications with increased transmission capacity and efficient spectral usage^17–36^.

Remarkably, for all of the above OAM-enabled extensive applications, the generation of OAM-carrying twisted light is of great importance. Various techniques for generating OAM-carrying twisted light beams have been proposed and demonstrated over the past years^6–8,18–20^. In general, there are two kinds of techniques, i.e. external beam shaping and internal beam lasing. The external beam shaping technique usually transforms a Gaussian beam into an OAM-carrying twisted light beam, e.g. computer-generated hologram^7,37^, mode converte^38^, spiral phase plate^39^, inhomogeneous anisotropic media^40^, fiber^41^, metamaterials^42–47^ and photonic integrated devices^48,49^. These tehniques relying on external beam shaping, however, suffer from relatively low conversion efficiency, degradation of beam quality, high power handling limitation, and added complexity with extra bulky mode conversion devices. Alternatively, using internal beam lasing technique, OAM-carrying twisted light can be also generated inside a laser resonator (i.e. twisted light laser), which may overcome some drawbacks of external beam shaping technique. To date there have been lots of prior seminal works related to OAM-carrying twisted light laser and an increasing interest has been gained to control light’s helicty at the source (i.e. OAM states from lasers)^50^. For instance, (1) Laguerre-Gaussian (LG) modes were generated in Nd:YAG lasers^51,52^; (2) LG modes seletection was demonstrated in diode-pumped solid-state lasers^53^; (3) a doughnut laser beam was generated as an incoherent superposition of two petal beams^54^; (4) high-order mode excitation was demonstrated in end-pumped solid-state lasers^55^; (5) the controlled generation of higher-order Poincaré sphere beams was demonstrated from a laser^56^; (6) OAM microlaser was fabricated and demonstrated on the InGaAsP/InP platform^57^. These works showed impressive lasing performance. Beyond the basic realization of OAM-carrying twisted light laser, the functionality and robustness are also worth taking full consideration. For a robust OAM-carrying twisted light laser, one would expect reconfigurable OAM and tunable wavelength. Fortunately, several approaches have been reported to add OAM reconfigurability or wavelength tunability to the twisted light laser. For example, (1) a digital laser for reconfigurable on-demand laser modes was demonstrated by using a phase-only spatial light modulator (SLM) as the back optical element of the cavity^58^; (2) a tunable midinfrared (6.0–12.5 μm) vortex laser was demonstrated by rotating the cascaded KTP crystals^59^. Wavelength-versatile optical vortex lasers were also reported^60^. In the demonstrated reconfigurable digital laser and wavelength-tunable optical vortex laser, free-space resonator cavities were employed. Very recently, all-fiber based resonator cavity was also employed for generating optical vortex beams^61,62^. However, the achievable OAM modes (e.g. OAM_±1_) were limited by the few-mode fiber. The generation of high-order OAM modes is full of challenge. Actually, the resonator cavity can be also constructed by a hybrid configuration, i.e. the combined free-space and fiber resonator cavity. For instance, a low-noise stretched-pulse Yb^3+^-doped fiber laser was demonstrated using free-space resonator cavity incorporating a piece of Yb^3+^-doped fiber as the gain medium^63^. Using fiber as the gain medium to form free-space to fiber resonator cavities can add enhanced flexibility to the laser configuration, i.e. one can have more choices on either bulky gain medium or fiber-doped gain medium for the desired lasing wavelength range. For example, for the potential application of twisted light in optical communications, twisted light laser in the 1.55 μm wavelength range is highly desired. In such case, a piece of erbium-doped fiber (EDF), commonly used in the communication band fiber laser, is preferred. Meanwhile, the all-fiber approach has great limitation in the achievable OAM modes. Hence, a possible approach could be to incorporate the EDF inside a free-space resonator cavity. The EDF serves as the gain medium for lasing in the 1.55 μm wavelength range, and the free-space resonator cavity allows more achievable OAM modes (high-order OAM modes). Remarkably, although most of the previously demonstrated twisted light lasers on various platforms (free space, fiber, chip) with favorable performance might achieve either high-order modes, or mode reconfigurability, or wavelength tunability, simultaneous implementation of all of these functions is still challengeable. To meet the increasing demand on a diverse of OAM-enabled applications with variable OAM values and wavelengths, robust twisted light laser with achievable high-order OAM modes, OAM reconfigurability and wavelength tunability is highly desired. In this scenario, a laudable goal would be to develop an OAM reconfigurable and wavelength tunable twisted light laser.

In this article, we propose and demonstrate a twisted light laser with achievable high-order OAM modes, OAM reconfigurability and wavelength tunability. The ring resonator of the twisted light laser is built upon a hybrid free-space and fiber platform. SLMs and a bandpass filter (BPF) are inserted into the laser resonator. Twisted light laser with reconfigurable OAM value from −10 to 10 and tunable wavelength from 1530 to 1565 nm (C band) is demonstrated in the experiment.

## Results

### Concept and principle

In general, a Fabry-Perot (FP) cavity formed by two mirrors is employed to construct a laser^64–66^. For a laser with cavity that is cylindrically symmetric, the lasing beam profile is also circularly symmetric often best solved using the Laguerre-Gaussian (LG) modal decomposition. The lasing beam functions can be written in cylindrical coordinates using generalized Laguerre polynomials. Taking the OAM-carrying LG beam laser as an example, the electrical field of a typical LG beam at a distance *z* from the waist in the paraxial approximation can be expressed as^7^

1 $$\begin{array}{c}{E}_{p\ell }(\rho ,\phi ,z)=\sqrt{\frac{2p!}{\pi (p\,+|\ell |)!}}\frac{1}{w(z)}{(\frac{\sqrt{2}\rho }{w(z)})}^{|\ell |}\exp (\frac{-{\rho }^{2}}{{w}^{2}(z)}){L}_{p}^{|\ell |}(\frac{2{\rho }^{2}}{{w}^{2}(z)})\\ \,\,\,\,\,\,\,\,\,\,\,\,\,\,\,\,\,\,\,\,\,\,\,\,\,\,\,\,\,\,\,\exp (i\ell \phi )\exp (ikz)\exp (\frac{ik{\rho }^{2}z}{2({z}^{2}+{z}_{R}^{2})})\exp (-i(2p\,+|\ell |+\,1){\tan }^{-1}(\frac{z}{{z}_{R}}))\end{array}$$

where *w*(*z*) = *w*_0_[(*z*^2^ + *z*_*R*_^2^)/*z*_*R*_^2^]^1/2^ is the 1/*e* radius of the LG beam at distance *z* from the beam waist *w*_0_, *ρ* is the radial distance from the beam center, *φ* is the azimuthal angle, *l* is the topological charge, *z*_*R*_ is the Rayleigh range, *L*_*p*_^*|l|*^ is the generalized Laguerre polynomial, *k* = 2*π*/*λ* is the wavenumber, *λ* is the wavelength, and (2*p* + |*l*| + 1)tan^−1^(*z*/*z*_*R*_) is the Gouy phase. Remarkably, *LG*_*0*_,_+*l*_ with right-handed helical phase trajectory and *LG*_*0*_,_*−l*_ with left-handed phase trajectory have the same spatial intensity distribution according to Eq. (1). Taking the typical FP cavity laser as an example, the scalar instantaneous electric field for *LG*_*0*_,_+*l*_ and *LG*_*0*_,_*−l*_ propagating in the forward direction can be written by

2 $${E}_{f}=u(r)\cos (kz-\omega t\pm \ell \phi )$$

where *w* is the angular frequency and *u*(*r*) is a complex function denoting the amplitude of the electric field. After the reflection by a cavity mirror, the scalar instantaneous electric field for *LG*_*0*_,_+*l*_ and *LG*_*0*_,_*−l*_ propagating in the backward direction can be expressed as

3 $${E}_{b}=u(r)\cos (-kz-\omega t\mp \ell \phi )$$

The superposition of forward and backward propagating electric fields gives the standing-wave electric field distribution for *LG*_*0*_,_+*l*_ and *LG*_*0*_,_*−l*_ as follows

4 $${E}_{\pm \ell }=2u(r)\cos (\omega t)\cos (kz\pm \ell \phi )$$

As a result, the instantaneous standing-wave intensity distribution is expressed as

5 $${I}_{\pm \ell }=4{|u(r)|}^{2}{\cos }^{2}(\omega t){\cos }^{2}(kz\pm \ell \phi )$$

From Eq. (5), one can clearly see that in a traditional LG beam laser with two mirrors forming an FP cavity, *LG*_*0*_,_+*l*_ and *LG*_*0*_,_*−l*_ beams have 2|*l*|-lobed transverse intensity distribution and always exist simultaneously. Note that although the forward and backward propagating electric fields overalp at a plane inside the cavity, the actual output even from a FP cavity is only the traveling wave. In particular, for a unidirectional ring resonator configuration, the electric field for *LG*_*0*_,_+*l*_ and *LG*_*0*_,_*−l*_ propagating in the ring resonator can be expressed as

6 $${E}_{\pm \ell }=u(r)\exp (ikz-i\omega t\pm i\ell \phi )$$

The superposition of *LG*_*0*_,_+*l*_ and *LG*_*0*_,_*−l*_ (zero radial order and azimuthal order *l*) with different intermodal phase shift can be written by^54^

7 $${E}_{even}={E}_{-\ell }+{E}_{+\ell }=u(r)\exp (ikz-i\omega t)[\exp (-i\ell \phi )+\exp (i\ell \phi )]$$

8 $${E}_{odd}=i({E}_{-\ell }-{E}_{+\ell })=i\,u(r)\exp (ikz-i\omega t)[\exp (-i\ell \phi )-\exp (i\ell \phi )]$$

Eqs (7) and (8) give even and odd petal modes with 2|*l*|-lobed transverse intensity distributions expressed as

9 $${I}_{even}=4{|u(r)|}^{2}{\cos }^{2}(\ell \phi )$$

10 $${I}_{odd}=4{|u(r)|}^{2}{\sin }^{2}(\ell \phi )$$

From Eqs (6–10), one can understand the laser principle in another way. The phase only azimuthal terms are not real-valued functions. It is the even and odd petal modes that are the real solutions to the wave equation.

In order to realize either *LG*_*0*_,_+*l*_ or *LG*_*0*_,_*−l*_ lasing, one may break the degeneracy of a standard laser cavity. One possible way is to insert an extra carefully designed mode-selection element (MSE) into the cavity^64,66^. Using pump control to produce desired modes or developing micro-chip laser could avoid the use of MSE inside the cavity^51,52,57^. Alternatively, a unidirectional ring resonator configuration with determined OAM value assisted by intra-cavity SLMs could be considered to overcome the shortcoming of the traditional FP cavity based laser.

**Figure 1:**
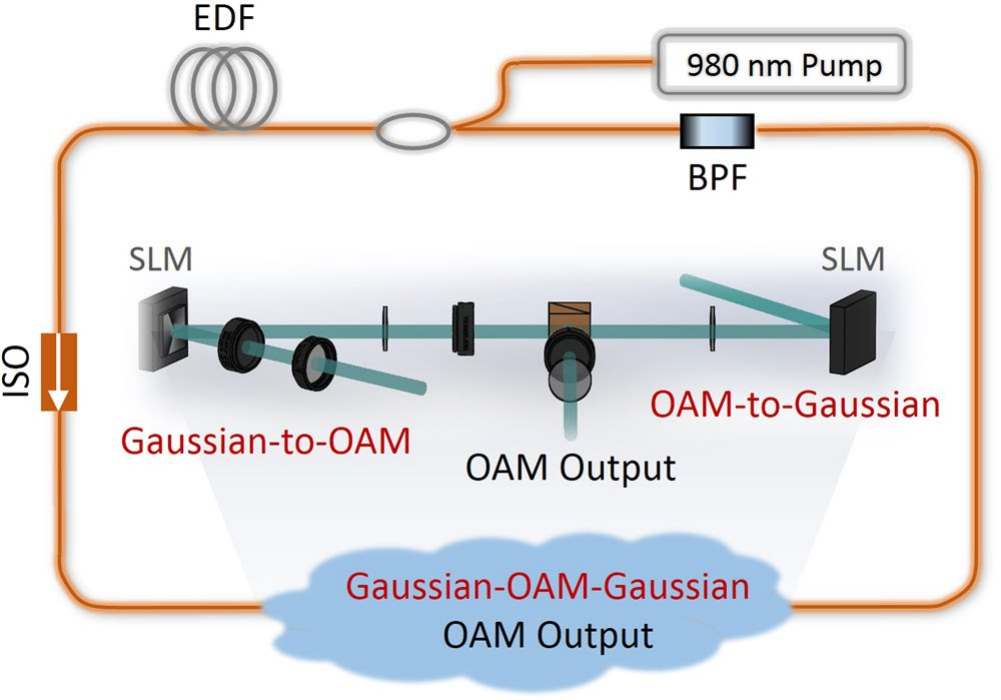
Concept and principle of the reconfigurable and tunable twisted light laser. It is a ring resonator configuration based on hybrid free-space and fiber platform. The gain medium of erbium doped fiber (EDF) pumped by a 980 nm laser enables lasing wavelength in the C band. An isolator (ISO) ensures unidirectional lasing. A Gaussian-OAM-Gaussian conversion module, including Gaussian-to-OAM and OAM-to-Gaussian by spatial light modulators (SLMs) and OAM output, supports determined reconfigurable OAM-carrying twisted light lasing. A tunable bandpass filter (BPF) enables wavelength tunable lasing.

The concept and schematic structure of the proposed reconfigurable and tunable twisted light laser is illustrated in Fig. 1. The gain medium of erbium-doped fiber (EDF) pumped with a 980 nm laser enables the lasing wavelength in the C band. For a ring resonator both clockwise (CW) and counterclockwise (CCW) propagation beams are supported. To ensure the unidirectional lasing, an isolator (ISO) is inserted into the ring resonator. To avoid simultaneous lasing of two OAM modes with opposite topological charges, a free-space Gaussian-OAM-Gaussian conversion module is inserted into the ring resonator. Hence, the twisted light laser shown in Fig. 1 is based on the hybrid free-space and fiber platform. The fiber part using fiber pigtailed components constructs the main ring resonator and provides the gain. The free-space part determines the desired OAM value of the twisted light for lasing. The Gaussian-OAM-Gaussian conversion module includes Gaussian-to-OAM conversion, OAM-to-Gaussian back conversion, and OAM output, as depicted in the inset of Fig. 1. Remarkably, the Gaussian-to-OAM conversion is enabled by an SLM loaded with a spiral phase pattern, simply expressed as

11 $$\varphi (r,\phi )=\exp (i\ell \phi )$$

The spiral phase pattern converts the planar phasefront of a Gaussian beam from fiber to the helical phasefront of an OAM-carrying twisted light beam. A part of the twisted light serves as the OAM output. The OAM-to-Gaussian back conversion is realized by another SLM loaded with an inverse spiral phase pattern written by

12 $$\varphi (r,\phi )=\exp (-i\ell \phi )$$

The inverse spiral phase pattern removes the helical phasefront of the OAM-carrying twisted light beam and back convert it to a Gaussian-like beam, which is further fed into the fiber. The key components are SLMs supporting determined reconfigurable OAM-carrying twisted light lasing. The OAM conversion from Gaussian beam and back conversion to Gaussian-like beam ensure the seamless connection between the fiber part and free-space part on the hybrid free-space and fiber platform. To realize the wavelength tunable lasing, a tunable bandpass filter (BPF) is inserted into the ring resonator. As a consequence, by properly switching the phase patterns loaded onto SLMs to determine the desired OAM value and adjusting the BPF to change the lasing wavelength, an OAM reconfigurable and wavelength tunable twisted light laser can be implemented using the modified ring resonator configuration shown in Fig. 1 based on the hybrid free-space and fiber platform.

### Experimental Configuration

**Figure 2:**
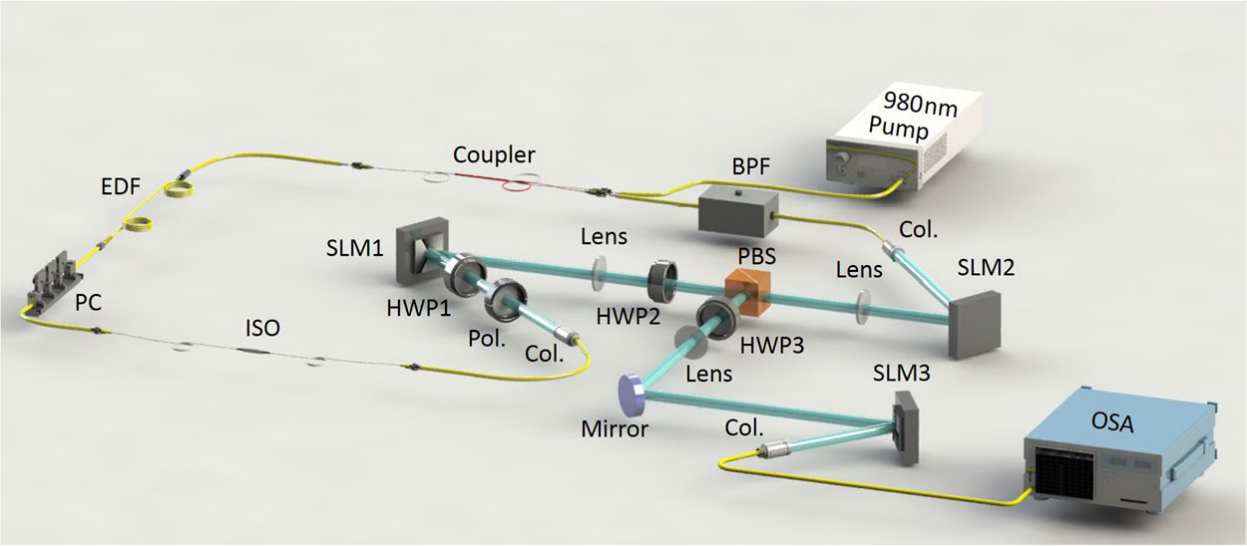
Experimental configuration of reconfigurable and tunable twisted light laser. EDF:erbium-doped fiber; PC: polarization controller; ISO: isolator; Col: collimator; Pol.: polarizer; HWP: half-wave plate; SLM: spatial light modulator; PBS: polarization beam splitter; BPF: bandpass filter; OSA: optical spectrum analyzer.

Figure 2 shows the experimental configuration of the reconfigurable and tunable twisted light laser. It has a ring resonator incorporating an EDF as the gain medium pumped by a 980 nm laser, an ISO for unidirectional lasing, a Gaussian-OAM-Gaussian conversion module for determined OAM reconfigurable operation and OAM output, and a tunable BPF for wavelength tunable operation. The 980 nm pump laser is fed into the ring resonator via a coupler. The gain medium of EDF supports lasing in the 1.55 μm band (C band). A polarization controlled (PC) is used to adjust the polarization to be aligned to the polarizer (Pol.) for optimized efficiency. An ISO ensures only CCW propagation inside the ring resonator. After a collimator (Col.), the collimated fiber output Gaussian beam passes through a Pol. and a half-wave plate (HWP1) before shining on the SLM1. Pol. ensures a pure linear polarization. HWP1 adjusts the polarization to be aligned to the working direction of the SLM for efficient generation of OAM beam. SLM1 is loaded with a spiral phase pattern for converting incident Gaussian beam to an OAM beam (Gaussian-to-OAM conversion). After SLM1, another HWP2 and a polarization beam splitter (PBS) are used to properly control the output power of the twisted light laser. The reflection port of PBS delivers the OAM output. The transmitted twisted light laser via the through port of PBS is sent to another SLM2 for OAM-to-Gaussian conversion. SLM2 loaded with another phase pattern converts the OAM beam back to a Gaussian-like beam, which is coupled back to the fiber. The followed tunable BPF enables the changeable lasing wavelength. The output of BPF is connected to the coupler together with the 980 nm pump laser for constructing the ring resonator. The intensity profile of the output OAM-carrying twisted light laser is observed by a camera not shown in Fig. 2. The OAM values of twisted light can be determined by its interferogram (interference with a reference Gaussian beam) or back conversion to a Gaussian-like beam by a third SLM3 loaded with the detection phase pattern. An optical spectrum analyzer (OSA) is used to observe the spectra. With proper update of phase patterns loaded onto SLMs and adjustment of BPF, OAM reconfigurable and wavelength tunable twisted light laser is achievable.

## Results

**Figure 3:**
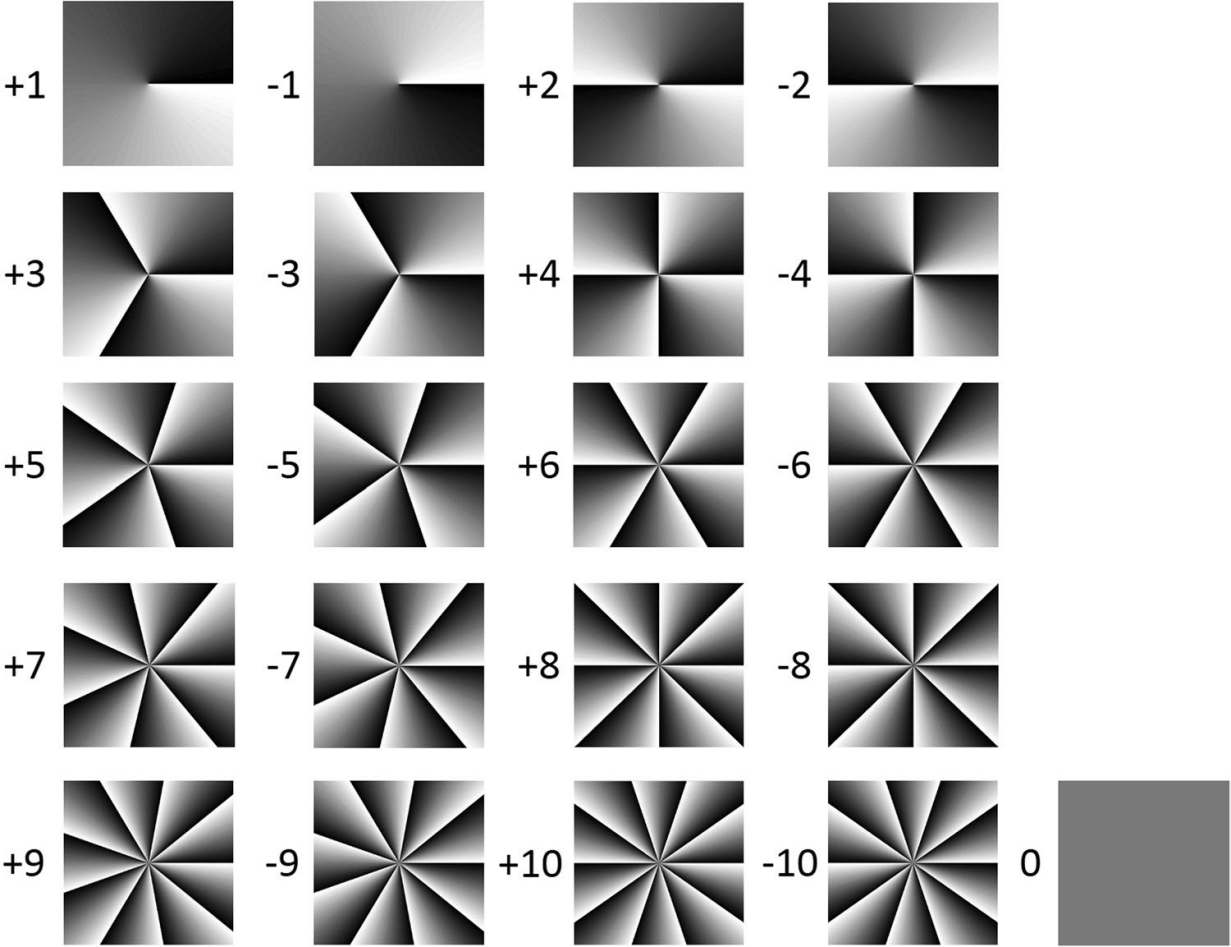
Spiral phase patterns loaded onto SLMs.

**Figure 4:**
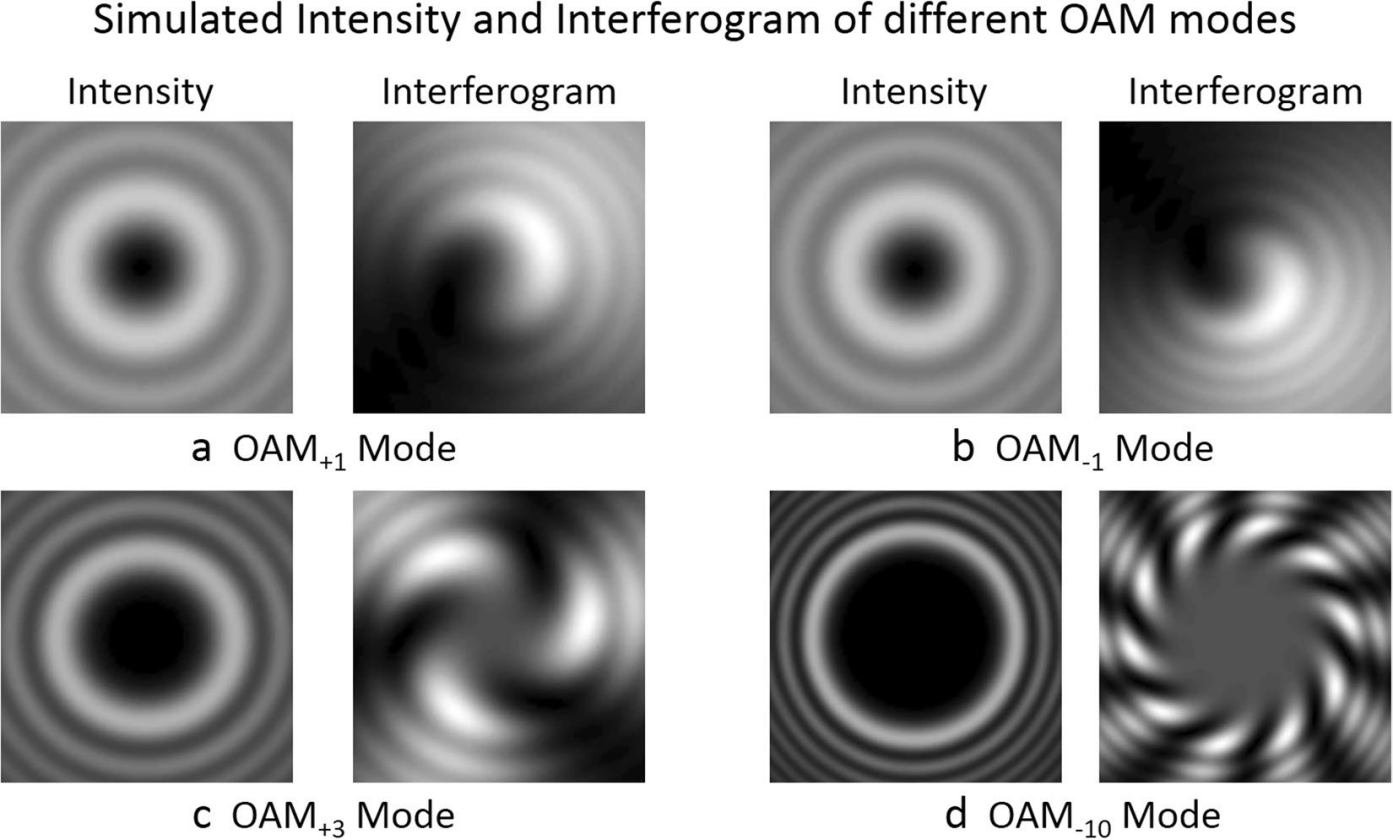
Simulated intensity and interferogram of different OAM modes. (**a**) OAM_+1_ mode. (**b**) OAM_−1_ mode. (**c**) OAM_+3_ mode. (**d**) OAM_−10_ mode.

In order to realize OAM reconfigurable twisted light laser (OAM value: −10 to 10) in the following experiment, we prepare corresponding spiral phase patterns to be switchably loaded onto the SLMs, which are shown in Fig. 3. We also simulate the generation of different OAM modes during the Gaussian-to-OAM conversion process shown in the inset of Fig. 1. Figure 4 shows the simulated intensity and interferogram of different OAM modes. One can see the generated OAM beams appear to be hypergemetric (with many rings). This might be explained with the fact that directly adding a spiral phasefront to a Gaussian beam gives an OAM beam but not a perfect OAM-carrying LG mode (zero radial order and azimuthal order *l*). Nevertheless, such OAM beams using straightforward and easy generation method and having all typical properties of OAM, are also widely used in different applications^6–9,17–20,22^. The interferograms (number of twists and twist direction) confirm the helical phase structures (OAM properties) of OAM beams.

**Figure 5:**
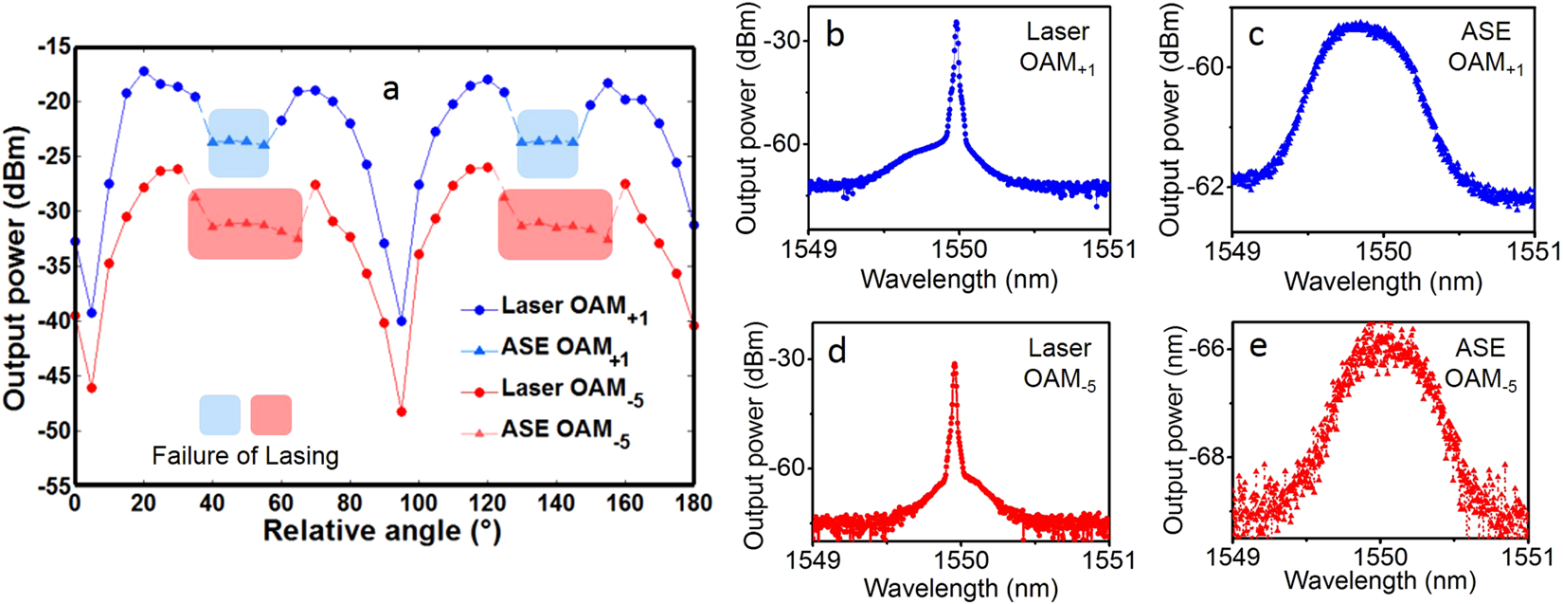
Measured results for flexible output power adjustment and typical lasing and ASE noise spectra. (**a**) Output power of twisted light laser versus relative angle between the HWP2 and the through port polarization direction of PBS in Fig. 2. Shadow regions: failure of lasing (ASE noise output). (**b**) Lasing spectrum for OAM_+1_. (**c**) ASE noise spectrum for OAM_+1_. (**d**) Lasing spectrum for OAM_−5_. (**e**) ASE noise spectrum for OAM_−5_. (**b**, **d**) Outside shadow regions in (a). (**c**,**e**) Within shadow regions in (**a**).

In the experimental configuration shown in Fig. 2, the combined use of HWP2 and PBS facilitates flexible output power adjustment by properly changing the relative angle between HWP2 and PBS. In the experiment, we first adjust the HWP2 to get the lowest output power and record its position as the start angle of 0 degree between the HWP2 and the through port polarization direction of PBS. Figure 5(a) shows measured output power for OAM_+1_ and OAM_−5_ as a function of the relative angle between the HWP2 and the through port polarization direction of PBS. With the increase of the relative angle, the output power increases first and then decreases. Note that when HWP2 rotates by α degree, the output light rotates by 2α degree compared to the incident light. Hence, one can clearly see the periodical behavior in Fig. 5(a) with a period of 90 degree. In particular, it is expected to achieve the maximum output power under a relatively angle of 45 degree when the HWP2 outputs light polarization aligned to the reflection port polarization direction of PBS. However, such maximum output power is not observed in Fig. 5(a), but a drop of output power is obtained instead. Such interesting phenomena can be briefly explained as follows. When adjusting the HWP2 and increasing the output power, the loss introduced in the ring resonator also increases. The maximum output power corresponds to the minimum power left in the ring resonator, leading to the failure of lasing. The shadow regions in Fig. 5(a) correspond to the amplified spontaneous emission (ASE) noise output, which should be avoided for the twisted light laser. To verify the laser output and ASE noise output, we measure the typical spectra outside and within the shadow regions, as shown in Fig. 5(b,c) for OAM_+1_ and Fig. 5(d,e) for OAM_−5_. One can clearly see the narrow lasing spectra in Fig. 5(b,d) and broad ASE noise spectra (limited by the filtering shape of BPF) in Fig. 5(c,e).

**Figure 6:**
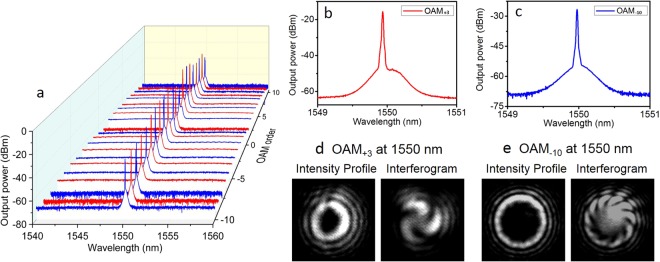
Measured results for OAM reconfigurable twisted light laser. (**a**) Measured spectra for twisted light laser with variable OAM values from −10 to 10. The lasing wavelength is 1550 nm. (**b**, **c**) Enlarged spectra for OAM_+3_ and OAM_−10_. (**d**,**e**) Measured intensity profiles and interferograms for OAM_+3_ and OAM_−10_. (**b**,**d**) OAM_+3_. (**c**,**e**) OAM_−10_.

We first demonstrate OAM reconfigurable twisted light laser. As shown in Fig. 6(a), the OAM value is varied from −10 to 10 simply by switching the order of spiral phase patten loaded onto SLMs. The lasing wavelength is 1550 nm. The typical enlarged spectra for OAM_+3_ and OAM_−10_ are depicted in Fig. 6(b,c), respectively. To confirm the successful lasing output of twisted light, we observe the intensity profiles and interferograms of twisted light by a camera, as shown in Fig. 6(d,e). The intensity profiles of OAM beams have doughnut shape with null intensity at the beam center due to phase singularity. The radius of the doughnut shape also increases with the order of OAM beam. One can also observe hypergeometric intensity profiles (multiple rings), which agree with the simulation results shown in Fig. 4. In the interferograms, the numer of twists and the twist direction indicate the magnitude of sign of OAM, respectively. From Fig. 6(d,e) one can confirm the successful output of OAM_+3_ and OAM_−10_ carrying twisted light laser.

**Figure 7:**
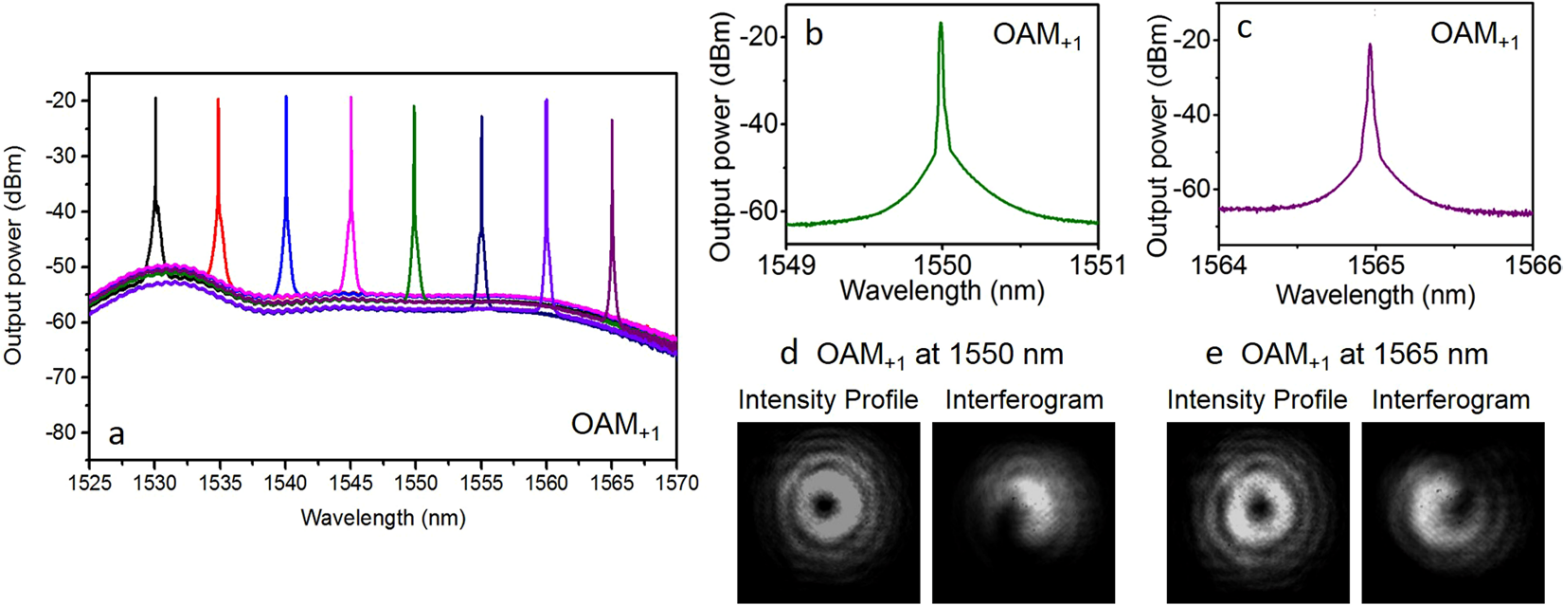
Measured results for wavelength tunable twisted light laser. (**a**) Measured spectra for twisted light laser with tunable wavelength from 1530 to 1565 nm. The twisted light laser is OAM_+1_. (**b**,**c**) Enlarged spectra for 1550 and 1565 nm. (**d**,**e**) Measured intensity profiles and interferograms for 1550 and 1565 nm. (**b**,**d**) 1550 nm. (**c**,**e**) 1565 nm.

We then demonstrate wavelength tunable twisted light laser. As shown in Fig. 7(a), the lasing wavelength is changed from 1530 to 1565 nm simply by adjusting the tunable BPF in the ring resonator. The twisted light laser is OAM_+1_. The typical enlarged spectra for 1550 nm and 1565 nm are depicted in Fig. 7(b,c), respectively. The recorded intensity profiles and interferograms of twisted light OAM_+1_ at 1550 nm and 1565 nm are shown in Fig. 7(d,e), respectively.

**Figure 8:**
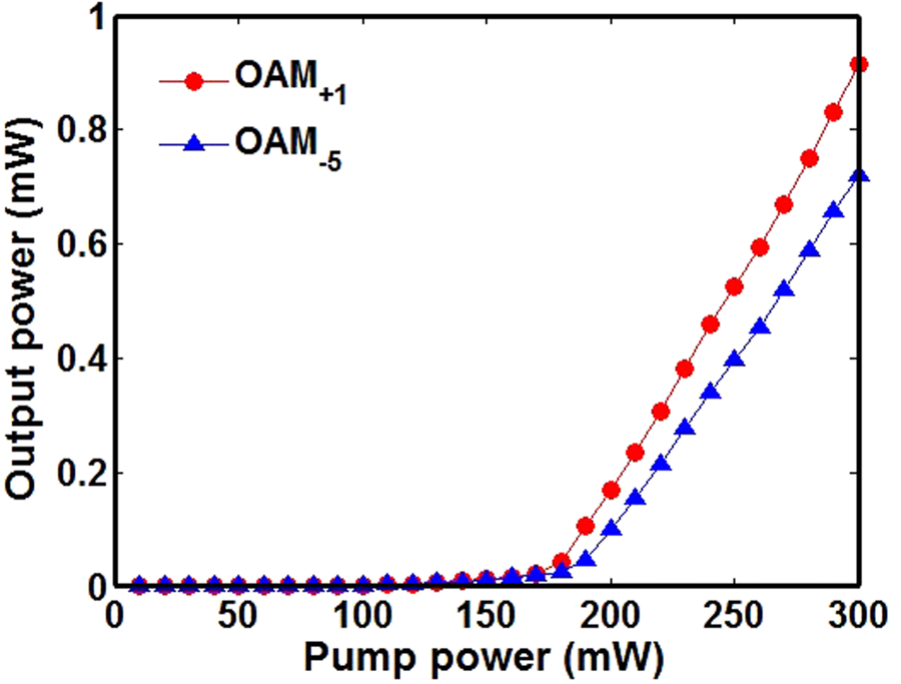
Measured output power of twisted light laser versus pump power for OAM_+1_ and OAM_−5_.

We further study the threshold of the twisted light laser. Figure 8 plots measured output power of twisted light laser as a function of the pump power for OAM_+1_ and OAM_−5_. It is shown that the twisted light laser has a lasing threshold of ~180 mW.

The obtained results shown in Figs 4–8 indiciate the successful implementation of OAM reconfigurable and wavelength tunable twisted light laser with favorable operation performance.

## Discussion

In summary, we design and demonstrate a reconfigurable and tunable OAM-carrying twisted light laser on a hybrid free-space and fiber platform. Instead of using traditional FP cavity, a ring resonator structure is employed. It consists of an EDF serving as the gain medium, a 980 nm pump laser, an ISO ensuring unidirectional lasing, a Gaussian-OAM-Gaussian conversion module supporting determined OAM reconfigurable operation and OAM output, and a tunable BPF enabling wavelength tunable operation. We study in detail the flexible output power adjustment, OAM reconfigurable twisted light laser, wavelength tunable twisted light laser, and the lasing threshold. In the experiment, twisted light laser with reconfigurable OAM from −10 to 10 and tunable wavelength from 1530 to 1565 nm covering the C band is demonstrated. The lasing threshold is measured to be ~180 mW (980 nm pump power).

The demonstrated reconfigurable and tunable twisted light laser features two distinct features as follows.

1. The hybrid free-space and fiber platform provides added flexibility to construct the laser configuration, offering more choices on either bulky or fiber-doped gain medium for the desired lasing wavelength range.
2. Simultaneous high-order OAM modes, OAM reconfigurability and wavelength tunability are realized by incorporating SLMs and BPF inside the ring cavity, which are challengeable in previous demonstrations.

Because of the above two advantages, the presented twisted light laser shows successful lasing of high-order OAM modes, reconfigurable OAM (−10 to 10) and tunable wavelength (1530 to 1565 nm) in the optical communication band (C band), which has not yet been demonstrated before.

Remarkably, the demonstrated twisted light laser also has several aspects worth improving.

1. The generated OAM beams appear to be hypergeometric with multiple rings as shown in both simulations and experiments. Although the OAM properties are still preserved and already widely used in many applications, one would expect to obtain perfect OAM modes such as LG modes. Fortunately, LG mode generation using SLMs has already been demonstrated^67–69^, which might be used for future performance improvement of the mode quality.
2. The constructed ring cavity resonator could lead to longitudinal modes instability for many cases (e.g. mechanical vibrations, thermal influences), which might become a potential cause for performance degradation of the twisted light laser. To overcome the longitudinal modes instability, several approaches have been proposed in the past decade. Recently, the combination of self-injection locking and stimulated thermal Rayleigh scattering was reported to enable longitudinal modes stability^70,71^, which might be used for future performance improvement of the mode stability.

In addition to the demonstrated continuous wave twisted light laser, other types of lasers such as single-longitudinal-mode twisted light laser and mode-locked fs pulsed twisted light laser could be considered with future improvement.

The demonstrations, indicating successful realization of reconfigurable and tunable twisted light laser, may further find a diverse of applications relying on flexible and robust generation of OAM-carrying twisted light beams. The hybrid free-space and fiber platform forming a laser resonator cavity with enhanced flexibility might be further developed to enable more general structured light laser.
